# Psychometric Analysis of an Academic Self-Attribution Questionnaire in Middle and High School Students in Italy: Implications of Gender and Age

**DOI:** 10.3390/ijerph20032235

**Published:** 2023-01-27

**Authors:** Eduardo Maria Moscato, Ana Isabel Obregón-Cuesta, María José Zapatero-Moreno, Jerónimo J. González-Bernal, Jessica Fernández-Solana, Luis Alberto Mínguez-Mínguez, Benito León-del-Barco, Santiago Mendo-Lázaro, Josefa González-Santos

**Affiliations:** 1 Department of Health Sciences, University of Burgos, 09001 Burgos, Spain; 2 Department of Mathematics and Computing, University of Burgos, 09001 Burgos, Spain; 3 Department of Education Sciences, University of Burgos, 09001 Burgos, Spain; 4 Department of Psychology and Anthropology, University of Extremadura, 10071 Caceres, Spain

**Keywords:** educational context, attributional styles, students, middle and high school, gender

## Abstract

This research aimed to validate the Academic Success and Failure Attribution Questionnaire (ASFAQ) and analyze gender and age differences in middle and high school students in Italy. Methods: From the ASFAQ questionnaire validated with Spanish students, an analysis of the psychometric characteristics of the scale was carried out using a Confirmatory Factor Analysis (CFA). To compare ASFAQ scores by gender and school year, the independent samples parametric t-test and Pearson’s coincidence test were used. (3) Results: in total, 976 students participated in the research, of which 515 were middle school students and 461 were high school students. The results showed a validity of the ASFAQ for Italian students, in addition to statistically significant differences between males and females, and school year. (4) Conclusions: The ASFAQ is reliable and valid to assess the attributional styles of academic success and failure in an Italian context. There are significant differences in sex and school year, and a consequence with age

## 1. Introduction

Individuals seek cause and effect relationships to explain their cognitive actions and behaviours, giving rise to attribution styles [1,2].

According to Piaget, the attribution process is rational. A later study and research on attribution proved otherwise [3]. There are, however, some contexts, such as education, where the same causes prevail, which helps us understand the origins of attributions [4]. 

Based on the studies of Heider and Kelly, Weiner attempts to understand the causes of success and failure in school performance. Causal perception varies according to gender, age, context, group or culture, generating different attribution styles: A) external or situational; B) internal, personal or dispositional [4,5]. Several studies have been conducted on this subject, and all agree that academic success or failure is closely related to internal and controllable factors, such as effort and ability, as well as external factors, such as luck and task difficulty [6,7,8]. Each attributional style supports or hinders learning by determining the motivation with which students approach academic tasks, and how this also influences their self-perception and even their performance [9].

There has been evidence that attributions are related to variables such as anxiety and depression, or school performance and self-efficacy, and that they are important determinants of students’ learning and self-esteem [10,11,12]. The stage of development at which secondary school students are and how they attribute the causes of their success and failure in school has important implications for growth [13,14]. Those students who attribute their achievements only to external causes will put little effort into learning and growing, whereas those who attribute them to a lack of intellectual ability will be severely affected in terms of confidence and academic performance [15,16]. In the same way, a person who associates success with his efforts or abilities is likely to feel pride and motivated to keep improving. In children, however, changes in causal attributions in problem solving are the result of metacognitive development, which affects not only their emotional reactions, but also their task orientation [15]. It has been shown that, although gender plays an important role in the attributional patterns used by students in the learning context, a consistent attributional tendency cannot be established, as it may vary depending on the school year and academic content area [2,16,17]. To promote adaptive attributional styles, there is a need for valid and reliable assessment instruments that are aimed at specific population groups that take into account the relevance of this aspect in the school context. 

There are several scales that focus on the academic context at the university level, such as the Attributional Style Questionnaire [18], the Multidimensional–multiattributional Causality Scale [19], the Sydney Attribution Scale [20], the Multidimensional Attribution Scale [21], the General Achievement Attributional Motivation Attributional Motivation Scale [22], and the Academic Attributional Style Questionnaire (AASQ) [23]. In the case of adolescent students, there is an adaptation of the Strategy and Attribution Questionnaire [24]; however, one of the most commonly used is the Locus Of Control scale (LOC), designed by Rotter, to measure students’ attributional style using 29 items assessing individual differences in generalized external and internal control expectancy. [25]. We also found the Academic Success and Failure Attribution Questionnaire (ASFAQ) [26], which specifically measures the attribution of academic success and failure of primary and secondary school students. None of these are validated in this specific population in Italy.

Likewise, the relevance of this study lies in the need to develop an instrument that considers the school context and assesses how students attribute the causes of academic success and failure to this specific population of Italian students. The main objective proposed, therefore, is to verify that the ASFAQ, which has shown good internal consistency in previous studies [26], is a rigorous scientific instrument with adequate validity and reliability to assess attributions of academic success and failure in this population. The factor structure needs to be confirmed in order to obtain evidence of validity and to be able to generalise the use of this questionnaire to more population groups. This is in addition to studying gender and school cycle differences in order to provide current and consistent empirical evidence.

## 2. Materials and Methods

### 2.1. Participants

The sample consisted of 976 students, of whom 515 were in the first (*n* = 163), second (n = 187) and third (n = 165) years of middle school, and 461 were in the first (n = 101), second (n = 85), third (n = 125), fourth (n = 84) and fifth (n = 66) years of high school. Overall, 399 subjects were male and 577 female, aged between 10 and 19 years. They were collected in 3 different public schools in the city of Gela, in the province of Caltanissetta.

### 2.2. Instruments

The Academic Success and Failure Attribution Scale is developed from Weiner’s theory of attributions [4,5]. The scale is composed of 24 items that score from 1 to 5, with 1 not agreeing at all and 5 strongly agreeing, measuring the attributions of academic success (12 items) and failure (12 items). Students attribute success or failure at school according to four elements: ability, effort, the difficulty of the task and luck. These are classified into three dimensions: locus of causality, stability and controllability. Regarding the locus of causality, it can be exogenous, i.e., associated with chance and/or task difficulty, or internal, associated with skill and effort. Moreover, its causes can be stable (ability) or unstable (effort and/or luck) [27,28].

ASFAQ consists of 4 items of controllable internal attributions related to academic success, 4 items of uncontrollable internal attributions related to academic success, 4 items of external attributions related to academic success, 4 items of controllable internal attributions related to academic failure, 4 items of uncontrollable internal attributions related to academic failure and 4 items of external attributions related to academic failure.

The choice of items in the Spanish version of ASFAQ was made by expert researchers based on an exhaustive study of Weiner’s theory of attributions in educational contexts [5].

A content validity of the ASFAQ questionnaire was carried out to ensure that the instrument covers the full range of dimensions related to the attributions of academic success and failure, through a panel of experts in education formed by teachers, pedagogues, and psychologists.

After this, for the construction and analysis of the psychometric characteristics of the scales, an exploratory factor analysis (EFA) was performed by Obregón-Cuesta et al. [26],

#### Instruments Translation

To translate the ASFAQ scale from Spanish to Italian, the following steps were followed:

- Step 1. In order to determine whether the questionnaire can be translated into Spanish, two bilingual experts reviewed the document in Spanish.
- Step 2. A translation of the questionnaire into Italian was completed by researchers and authors.
- Step 3. It was reviewed by the experts and two Italian monolingual researchers, and the necessary modifications were made where necessary
- Step 4. After translating the scale or questionnaire into Spanish, bilingual experts ensured that it is was faithful as possible to the original Spanish version by verifying its agreement and coherence with the translations of the same authors and/or researchers.

### 2.3. Procedure

First of all, the head teachers at the schools where the research was carried out were contacted, and the objectives of the research were explained. When the collaboration was accepted, the participants from the different classrooms signed the informed consent form to be able to participate, and the data were collected by providing the scales. These were filled in anonymously and the confidentiality of the data obtained was always guaranteed, always explaining that they were intended for research purposes. Data collection was carried out during school hours and all the necessary instructions were provided for correct completion. The questionnaires were completed in approximately 15 minutes, individually within the school environment, a suitable environment without distractions. The ethical guidelines of the American Psychological Association were followed throughout the procedure.

### 2.4. Data Analysis

The factor structure found in the ASFAQ scale, validated by Obregón-Cuesta et al. [26], was confirmed by a confirmatory factor analysis (CFA). The AMOS-21 program was used to perform the CFA.

The ASFAQ scores by gender were tested using a parametric t-test for independent samples, as well as by school year. The statistical significance value established was *p* < 0.05, using SPSS software version 25 (IBM-Inc, Chicago-IL-USA).

## 3. Results

### 3.1. Confirmatory Factor Analysis

Based on the fit indices used (χ2, χ2/gl, Comparative Fit Index (CFI), Tucker–Lewis Index (TLI), Root Mean Square Error of Approximation (RMSEA) and Standardized Root Mean-Square (SRMR)), applying the maximum likelihood method, three attribution models related to success and three attribution models related to failure were obtained. The χ2 had to acquire significant values *p* > 0.05 [29], χ2/gl was considered acceptable [30] when it was less than 5, and CFI and TLI were considered acceptable when the values were above 0.90 and ≥0.08 for RMSEA [31] y SRMR [32].

#### Confirmatory Factor Analysis of Success and Failure Attributions

The success and failure attribution models had a good fit, with a χ2/g.l value of less than 5, CFI and TLI values above 0.90, and lower RMSEA and SRMR values of less than 0.8 (Table 1).

**Figure 1:**
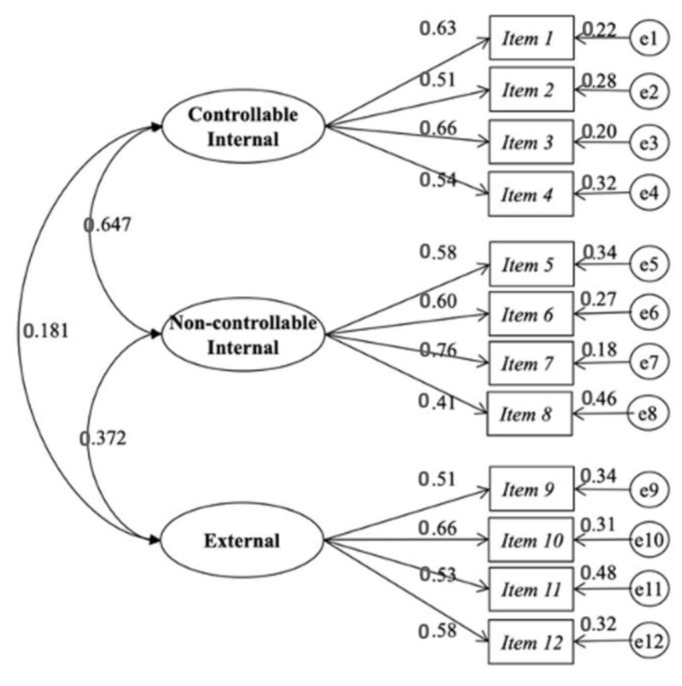
Model of three related factors of the Scale of Attributions of the Academic Success.

The t-values (range 8.68–10.72) of the unstandardised regression coefficients of the success attributions model were statistically significant. The range of the standardised coefficients for factor one (0.509–0.060), two (0.413–0.764) and three (0.530–0.658), demonstrated the consistency of the indicators for the measurement of the constructs, being clearly related (Figure 1).

**Figure 2:**
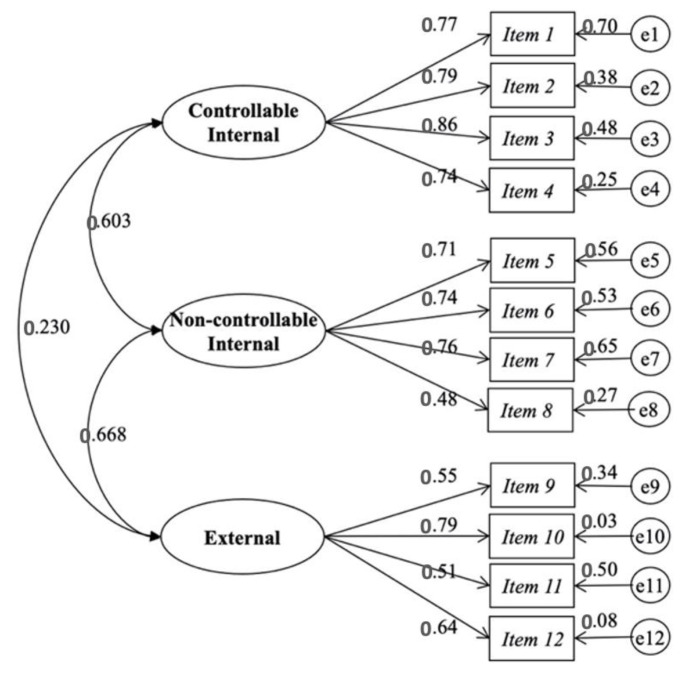
Model of three related factors of the Scale of Attributions of the Academic Failure.

Similarly, the t-values (range 7.14–19.90) of the unstandardized regression coefficients of the failure attributions model were statistically significant. The range of standardized coefficients for factor one (0.742–0.865), two (0.706–0.759) and three (0.451–0.791) were statistically significantly related, demonstrating the consistency of the indicators for the measurement of the constructs (Figure 2).

### 3.2. Analysis of ASFAQ According to Gender

All students showed a higher attribution of success to internal controllable causes, followed by internal non-controllable causes and finally external causes.

The results of the inferential analysis showed statistically significant differences in the attributions of academic success according to gender, but not in those of academic failure (Table 2). More specifically, the female participants demonstrated a greater attribution of their academic success to controllable internal causes (*p* = 0.015). No significant differences were found in the non-controllable internal attributions of academic success according to gender (*p* = 0.077), such as intelligence, good memory, talent, or calm character; this was also the case in external causes (*p* = 0.230), such as easy exams, good luck, low demands, or good explanations from teachers.

### 3.3. Analysis of ASFAQ According to the School Cycle

The results of the inferential analysis showed statistically significant differences in all attributions of academic success and in the external attribution of academic failure, depending on the school cycle (Table 3). More specifically, middle school students showed a higher attribution of their academic success to internal controllable causes (*p* < 0.001), internal uncontrollable causes (*p* < 0.001) and external causes (*p* < 0.001) than upper secondary students. On the other hand, the failure attribution scores were lower than success attribution scores.

In terms of attributions of academic failure, statistically significant differences were found in external attributions between middle and upper secondary students, with older students obtaining higher scores, attributing their failures to aspects such as bad luck or the difficulty of exams.

It is important to highlight this last aspect, since the most important difference is shown in external causes, being greater in older students. There is a statistically significant direct relationship between age and the external attribution of failure, in such a way that the older the students are, the higher the external attribution of academic failure is: r(974) = 0.342, *p* < 0.001.

## 4. Discussion

The main objective of the study was the validation of the ASFAQ in Italian secondary school students. It is important to have validated and targeted tools for this specific population in the school context, as the way in which students attribute the causes of their success and/or failure at school is of particular relevance to their development, academic performance and social context. [13,23,33,34,35].

Considering the results obtained, the questionnaire has proven to be an instrument with sufficient scientific rigour and adequate validity and reliability, which measures what it is intended to measure, i.e., the attributions of academic success and failure in secondary school students in Italy; this confirms the first hypothesis put forward. Likewise, it also confirms the hypothesis that was put forward about the differences attributable to gender and school cycle, providing up-to-date and highly consistent empirical evidence.

The confirmatory analysis revealed the adequate internal consistency and validity of the instrument. Likewise, the instrument proved to be suitable for the assessment of the attributions of academic success and failure in the sample of students analyzed, as it is also invariant by gender.

There is currently no specific instrument that analyses this aspect in the school context in secondary school students in Italy, even though the analysis of the attributional style of students at this fundamental stage of their development can be of great benefit. One study found that adolescents with anxiety were more likely to have negative interpretations, compared to those without anxiety; the former are more likely to develop maladaptive attributional styles that negatively influence attentional, cognitive, threat perception processing, and cause lower executive functioning or the presence of interpretative biases in a threatening social context [36,37,38,39]. These variables at the psycho-emotional level, such as anxiety or depression, can be associated with internal and stable attributional patterns when negative situations occur; in positive situations, they can be associated with external attributional patterns [40]. However, most of the studies conducted so far have been carried out in adults or the general population [24]. Furthermore, within the school context, they have focused mainly on university students, in which a statistically significant positive relationship was found between attributional styles, such as academic performance, academic self-concept and/or learning goal orientation [24,41,42,43,44].

As for the analysis by gender, the results of the study show that all students attribute their success to internal controllable causes, followed by internal non-controllable causes, as in the study by Almeida et al. [17], where students mainly appealed to internal causes for both their successes and failures at school. More specifically, it was the females in our study who attributed their academic success to internal controllable causes, although no differences were found in the internal non-controllable causes (intelligence, good memory, talent, or calm character), nor in the external ones (easy exams, good luck, low demands or good explanations from teachers) with respect to gender. However, another study shows that the attributional style of male students was more adaptive, attributing their successes to ability, through internal, stable and uncontrollable causes, and their failures to lack of effort, i.e. internal, unstable and controllable causes [45]; male students, therefore, tend to preserve self-concept and self-esteem. [17].

In addition to gender, other factors have been shown to have a significant effect on students’ academic attributions, such as age and academic cycle [16,46,47]. The results of our research showed statistically significant differences in all attributions of academic success and in the external attribution of academic failure. Middle school students attribute their academic success to a greater extent to both internal controllable, internal uncontrollable and external causes, compared to high school students. These results coincide with those found in the study by Obregón-Cuesta et al. [26], where internal and external causes were attributed by students in lower grades; this is also the case in the research of English et al. [45], where students in lower grades showed a mixed attributional pattern in comparison to higher grades.

However, the most important difference found in our results relates to the attribution of external causes to academic failure in upper secondary students compared to the rest, establishing a positive relationship. On the contrary, in the literature, the results found in several studies contradict this; in this case, students in lower grades are the ones who significantly attribute their academic results to external causes [46,48,49]. However, this is partially corroborated by other research, where upperclassmen were more likely to attribute their academic failures to internal controllable and external causes than lower grade students [26].

Therefore, the ASFAQ instrument is a good tool for assessing attributions of academic success and failure, as well as helping to monitor the attributional strategies and styles of secondary school students. Furthermore, the significant differences found with respect to gender and academic cycle reiterate the importance of considering these aspects in the educational environment [29]. 

In terms of the limitations of our study, it should be noted that the results cannot be generalised to the whole population worldwide, as the sample was taken only in Italy. It would be important to take into account the effect of culture when trying to extend the results to another population. The use of self-report questionnaires may also be a limitation in the research, as these questionnaires should be interpreted in a cautious way, despite demonstrating good internal consistency, validity and reliability for this population.

Another limitation is the lack of convergent and divergent validity that correlates the ASFAQ with other instruments of attribution measurement.

There is a need for further research in this area, as few studies have been carried out on this topic, specifically in middle and high school students. Taking into account the attributional style of students can be a turning point in school life, so it is suggested that more studies be carried out, with a large sample size and by collecting additional information.

In terms of strengths, a valid and reliable instrument measuring students’ attributions in Italy was obtained. Moreover, the significant differences between gender and school cycle provide important data, which may allow the targeting of more appropriate psychoeducational interventions.

## 5. Conclusions

This study can demonstrate the properties of the ASFAQ for the assessment of attributions of academic success and failure in secondary school students. For this reason, the present research provides a rigorous, scientific instrument with good validity and reliability for assessing attributional style in the educational context of secondary school. However, its role in promoting adaptive attitudes and behaviours to support student learning should be added. Likewise, the significant differences found in terms of gender and school cycle provide relevant and updated information to promote the development and implementation of psychoeducational interventions aimed at correcting the adaptive attributional style of secondary school students.

## Figures and Tables

**Table 1:** Goodness-of-fit indices of the proposed academic success and failure attribution models.

Models	χ2	CMIN/DF	CFI	TLI	RMSEA	SRMR
3 related factors Success	*p* < 0.001	2.000	0.948	0.933	0.044	0.050
3 related factors Failure	*p* < 0.001	2.791	0.949	0.934	0.059	0.058

CMIN = chi2 ratio over the degrees of freedom; CFI = comparative fit index; TLI = Tucker–Lewis index; RMSEA = root mean square error of approximation; SRMR = standardised residual root mean square.

**Table 2:** Results of the T-test for independent samples between ASFAQ and gender.

ASFAQ	Gender	N	Mean	STD	*p* Value
Controllable internal success	man	399	12.48	2.022	0.015
	woman	575	12.79	1.847	
Non-Controllable internal success	man	399	10.97	2.190	0.077
	woman	575	10.72	2.129	
External success	man	399	8.93	1.895	0.230
	woman	575	8.78	1.896	
Controllable internal failure	man	399	10.18	2.984	0.078
	woman	575	9.84	2.955	
Non-Controllable internal failure	man	399	8.42	2.537	0.060
	woman	575	8.72	2.278	
External failure	man	399	8.21	2.333	0.363
	woman	575	8.34	2.244	

STD = Standard Deviation.

**Table 3:** Results of the ANOVA test between ASFAQ and the school cycle.

	Secondary Cycle	N	Mean	STD	*p* Value
Controllable internal success	Middle School	514	13.05	1.807	<0.001
	High School	460	12.23	1.965	
Non-Controllable internal success	Middle School	514	11.19	2.023	<0.001
	High School	460	10.41	2.227	
External success	Middle School	514	9.36	1.741	<0.001
	High School	460	8.25	1.892	
Controllable internal failure	Middle School	514	9.96	3.103	0.882
	High School	460	9.99	2.818	
Non-Controllable internal failure	Middle School	514	8.54	2.280	0.418
	High School	460	8.67	2.509	
External failure	Middle School	514	7.56	2.082	<0.001
	High School	460	9.10	2.220	

STD = Standard Deviation.

## Data Availability

Not applicable.

## References

[1] Moè A., de Beni R. (2002). Stile attributivo, motivazione ad apprendere ed atteggiamento strategico. Una Rass. Psicol. Clin. Dello Svilupp..

[2] Rossi F. (2021). Differenze di genere nelle strategie di apprendimento e nella prospettiva temporale: Una ricerca esplorativa nella scuola secondaria di secondo grado. Orientamenti Pedagog..

[3] Forgas J.P. (1995). Comportamento Interpersonale.

[4] Weiner B., Sorrentino R., Higgins E. (1986). Attribution, Emotion, and Action. Handbook of Motivation and Cognition: Foundations of Social Behavior.

[5] Weiner B. (2010). Attribution Theory. The Corsini Encyclopedia of Psychology.

[6] Mok M.M.C., Kennedy K.J., Moore P.J. (2011). Academic attribution of secondary students: Gender, year level and achievement level. Educ. Psychol..

[7] Lam S.-F., Yim P.-S., Law J.S.F., Cheung R.W.Y. (2004). The effects of competition on achievement motivation in Chinese classrooms. Br. J. Educ. Psychol..

[8] Bruschi B. (2000). Elaborazione di un Questionario Sulla Motivazione e Sul Metodo di Studio per Gli Studenti delle Scuole Medie Superiori. Ph.D. Thesis.

[9] Fissi S. (2004). Pulsioni, affetti motivazioni, nella costruzione degli schemi relazionali interiorizzati e delle rappresentazioni del Sé e dell’oggetto. Studi Junghiani.

[10] Nerstad C.G.L., Roberts G.C., Richardsen A.M. (2013). Achieving success at work: Development and validation of the Motivational Climate at Work Questionnaire (MCWQ). J. Appl. Soc. Psychol..

[11] Zimmerman B.J., Kitsantas A. (1999). Acquiring writing revision skill: Shifting from process to outcome self-regulatory goals. J. Educ. Psychol..

[12] Murdaca A.M. (2014). Predizione della credenza di autoefficacia, dell’ansia e degli stili decisionali sui risultati universitari. Form. Insegn. Riv. Internazionale Di Sci. Dell’Educazione E Della Form..

[13] Lohbeck A., Grube D., Moschner B. (2017). Academic self-concept and causal attributions for success and failure amongst elementary school children. Int. J. Early Years Educ..

[14] Desbouts C.G. (2006). La Scuola Non Fa per Me.

[15] Mason L., Arcani S. (2001). Motivazione all’impegno scolastico, attribuzioni causali e rendimento in studenti di scuola media e superiore. Psicol. Clin. Dello Svilupp..

[16] Inglés C., Díaz-Herrero A., García-Fernández J., Ruiz-Esteban C. (2011). Gender and academic year as predictors of attributions in reading and mathematics in students of Compulsory Secondary Education. An. Psicol..

[17] Almeida L., Miranda L., Guisande M. (2008). Causal attributions for academic success and failure. Estud. Psicol..

[18] Peterson C., Semmel A., von Baeyer C., Abramson L.Y., Metalsky G.I., Seligman M.E.P. (1982). The attributional Style Questionnaire. Cogn. Ther. Res..

[19] Lefcourt H.M., von Baeyer C.L., Abramson L.Y., Metalsky G.I., Seligman M.E.P. (1982). The multidimensional-multiattributional causality scale: The development of a goal specific locus of control scale. Cogn. Ther. Res..

[20] Inglés C., Rodríguez-Marín J., González-Pienda J. (2008). Adaptation of the Sydney Attribution Scale in a Spanish college population. Psicothema.

[21] Tapia J. (2014). Causality attribution and achievement motivation. II: Evolutive study of attributions influence in manifest level of achievement motivation. Stud. Psychol..

[22] Durán-Aponte E., Pujol L. (2013). Attribution Scale of General Achievement Motivation (EAML-G): Adaptation and analysis of its psychometric properties. Estud. Pedagógicos.

[23] Peterson C., Barrett L. (1987). Explanatory Style and Academic Performance Among University Freshmen. J. Personal. Soc. Psychol..

[24] Heikkilä A., Niemivirta M., Nieminen J., Lonka K. (2010). Interrelations among university students’ approaches to learning, regulation of learning, and cognitive and attributional strategies: A person oriented approach. High. Educ..

[25] Rotter J. (1966). Generalized expectancies for internal versus external control of reinforcement. Psychol. Monogr..

[26] Obregón-Cuesta A.I., Rodríguez-Fernández P., León-Del-Barco B., Mendo-Lázaro S., Mínguez-Mínguez L.A., González-Santos J., González-Bernal J.J. (2022). Validation of an Academic Self-Attribution Questionnaire for Primary and Secondary School Students: Implications of Gender and Grade. Int. J. Environ. Res. Public Health.

[27] Ibarra Tancara J. (2019). Atribucional styles in the perception of academic achievement and interpersonal relations in pregrado students of psychology. Rev. Investig. Psicol..

[28] Weiner B. (2000). Attributional Thoughts about Consumer Behavior. J. Consum. Res..

[29] Chan L.K.S., Moore P.J. (2007). Development of Attributional Beliefs and Strategic Knowledge in Years 5–9: A longitudinal analysis. Educ. Psychol..

[30] Hu L., Bentler P. (1995). Evaluating model fit. Structural Equation Modeling: Concepts, Issues, and Applications.

[31] Browne M.W. (1993). Alternative Ways of Assessing Model Fit. Sociol. Methods Res..

[32] Hu L., Bentler P. (1999). Cutoff criteria for fit indexes in covariance structure analysis: Conventional criteria versus new alternatives. Struct. Equ. Model. A Multidiscip. J..

[33] Álvarez A., Suárez N., Tuero E., Núñez J.C., Valle A., Regueiro B. (2015). Family involvement, adolescent self-concept and academic achievement. Eur. J. Investig. Health Psychol. Educ..

[34] Fernández-Bustos J., González-Martí I., Contreras O., Cuevas R. (2015). Relationship between body image and physical self-concept in adolescent females. Rev. Latinoam. Psicol..

[35] Normandeau S., Gobeil A. (1998). A Developmental Perspective on Children’s Understanding of Causal Attributions in Achievement-related Situations. Int. J. Behav. Dev..

[36] Haller H., Lauche R., Cramer H., Rampp T., Saha F.J., Ostermann T., Dobos G. (2016). Craniosacral Therapy for the Treatment of Chronic Neck Pain: A Randomized Sham-controlled Trial. Clin. J. Pain.

[37] Blair K.S., Geraci M., Korelitz K., Otero M., Towbin K., Ernst M., Leibenluft E., Blair R., Pine D.S. (2011). The Pathology of Social Phobia is Independent of Developmental Changes in Face Processing. Am. J. Psychiatry.

[38] Delgado B., Aparisi D., García-Fernández J.M., Sanmartín R., Redondo J., Inglés C.J. (2018). Attributional styles in Spanish students of compulsory secondary education with high social anxiety self-reported. Rev. Latinoam. Psicol..

[39] Schlier B., Helbig-Lang S., Lincoln T.M. (2015). Anxious but Thoroughly Informed? No Jumping-to-Conclusions Bias in Social Anxiety Disorder. Cogn. Ther. Res..

[40] Moore D.W., Schultz N.R. (1983). Loneliness at adolescence: Correlates, attributions, and coping. J. Youth Adolesc..

[41] Albert M.A., Dahling J.J. (2016). Learning goal orientation and locus of control interact to predict academic self-concept and academic performance in college students. Personal. Individ. Differ..

[42] Mayora-Pernía C.A. (2015). Locus de control y rendimiento académico en educación universitaria: Una revisión bibliográfica. Rev. Electrónica Educ..

[43] Serrano Encinas D., Bojórquez-Díaz C., Vera Noriega J. Rendimiento Académico y Locus de Control en Estudiantes Presenciales y no Presenciales. Proceedings of the X Congreso Nacional de Investigación Educativa.

[44] Terán Vera M. (2018). Locus de control, motivación y rendimiento académico en estudiantes del primer ciclo de estudios generales de la universidad de San Martín de Porres. Ph.D. Thesis.

[45] Inglés C.J., Díaz-Herrero Á., García-Fernández J.M.J., Ruiz-Esteban C., Delgado B., Martínez-Monteagudo M.C.M. (2012). Gender and grade differences in students of secondary education. Rev. Latinoam. Psicol..

[46] Gonzaba L., Morais S., Santos J., Jesus S. Atribuições causais do sucesso e do fracasso académico: Estudo comparativo de estudantes do ensino secundário e do superior. Proceedings of the XI Conferência Internacional de Avaliação Psicológica: Formas e Contextos.

[47] Barca A., Peralbo M., Cadavid M. (2003). Atribuciones causales y rendimiento académico en alumnos de educación secundaria: Un estudio a partir de la subescala de atribuciones causales y multiatribucionales (EACM). Psicol. Teoría E Práctica.

[48] De la Torre C., Godoy A. (2003). Individual differences in the causal attributions of teachers and their influence on the affective component. Interam. J. Psychol..

[49] Boruchovith E. (2004). A study of causal attributions for success and failure in mathematics among Brazilian students. Interam. J. Psychol..

